# Limitations of Deuterium-Labelled Substrates for Quantifying NADPH Metabolism in Heterotrophic *Arabidopsis* Cell Cultures

**DOI:** 10.3390/metabo9100205

**Published:** 2019-09-28

**Authors:** Edward N. Smith, James S. O. McCullagh, R. George Ratcliffe, Nicholas J. Kruger

**Affiliations:** 1 Department of Plant Sciences, University of Oxford, Oxford OX1 3RB, UK; edward.smith@plants.ox.ac.uk; 2 Chemistry Research Laboratory, Department of Chemistry, University of Oxford, Oxford OX1 3TA, UK

**Keywords:** *Arabidopsis thaliana*, deuterium, flavin enzymes, flux analysis, NADPH, redox, water exchange

## Abstract

NADPH is the primary source of cellular reductant for biosynthesis, and strategies for increasing productivity via metabolic engineering need to take account of the requirement for reducing power. In plants, while the oxidative pentose phosphate pathway is the most direct route for NADPH production in heterotrophic tissues, there is increasing evidence that other pathways make significant contributions to redox balance. Deuterium-based isotopic labelling strategies have recently been developed to quantify the relative production of NADPH from different pathways in mammalian cells, but the application of these methods to plants has not been critically evaluated. In this study, LC-MS was used to measure deuterium incorporation into metabolites extracted from heterotrophic *Arabidopsis* cell cultures grown on [1-^2^H]glucose or D_2_O. The results show that a high rate of flavin-enzyme-catalysed water exchange obscures labelling of NADPH from deuterated substrates and that this exchange cannot be accurately accounted for due to exchange between triose- and hexose-phosphates. In addition, the duplication of NADPH generating reactions between subcellular compartments can confound analysis based on whole cell extracts. Understanding how the structure of the metabolic network affects the applicability of deuterium labelling methods is a prerequisite for development of more effective flux determination strategies, ensuring data are both quantitative and representative of endogenous biological processes.

## 1. Introduction

NADPH is the primary source of cellular reductant for both biosynthesis and protection from oxidative stress. In plants, NAD(P) coenzymes are synthesized de novo from aspartate as well as being recovered by salvage mechanisms [1,2]. Although reactions which consume NAD(H) and NADP(H) are important for plant development and stress responses [3], the role of these coenzymes in redox metabolism depends on their oxidation and reduction. The redox couples turn over rapidly with a half-life of <1 s [4], as many different reactions simultaneously contribute to their oxidation or reduction. In photosynthetic plant tissues, ferredoxin-NADP^+^ reductase transfers electrons from photosystem I via ferredoxin to NADP^+^_._ NADPH is also produced in all plant tissues by the oxidation of carbon substrates via enzymes including: glucose-6-phosphate dehydrogenase (G6PDH, EC 1.1.1.49), 6-phosphogluconate dehydrogenase (6PGDH, EC 1.1.1.44), NADP-isocitrate dehydrogenase (NADP-IDH, EC 1.1.1.42), NADP-malic enzyme (NADP-ME, EC 1.1.1.40), methylenetetrahydrofolate dehydrogenase (MTHFD, EC 1.5.1.5) [5] and non-phosphorylating glyceraldehyde 3-phosphate dehydrogenase (GAPDH, EC 1.2.1.9). NADPH can also be generated from NADH via the phosphorylation of NADH by NAD kinases using ATP [6,7,8]. It is presumed these diverse sources make a variable, but potentially significant contribution to NADPH production, and although this is supported by phenotypes of genetic knock-outs under certain conditions [9,10,11,12,13], the relative contributions of the different sources have yet to be determined.

The wide involvement of redox coenzymes in biosynthetic and maintenance processes means that engineering metabolism requires careful coenzyme balancing to ensure any modification does not detrimentally affect cellular redox state [14,15]. Numerous examples of redox balancing strategies exist from bacterial and yeast engineering [16,17,18,19,20,21,22], but a lack of quantitative understanding of redox metabolism in plants means that rational strategies have not been explored in depth in such systems. Modifying the supply of reducing power may aid strategies designed to improve photosynthetic productivity [23], nitrogen use efficiency [24], and yield in crops under stress [25]. For example, stress tolerance and resistance to *Phytophthora nicotianae* was improved in tobacco by increasing the capacity for cytosolic NADPH production by expressing a plastidic isoform of G6PDH in the cytosol [26].

Evidence from genetic knock-outs shows that multiple different sources of reducing power are important under various types of plant-stress conditions, but normal growth phenotypes under non-stress conditions show a redundancy in the supply of NADPH [9,10,11,12,13]. Carbon-based flux analysis has shown that the oxidative pentose phosphate pathway (oxPPP) cannot supply sufficient reductant for biosynthesis in heterotrophic cells and therefore other sources must make a significant contribution [27]. Furthermore, the oxPPP flux has been shown to vary independently of the reductant demand for biosynthesis [28], consistent with the idea that it is not the sole source of NADPH. However, carbon-based analysis cannot quantify NADPH flux from pathways where an identical carbon transformation can produce either NADPH or NADH. This is the case for malic enzyme (ME) and isocitrate dehydrogenase (IDH) and leads to the lack of empirical measurement of total NADPH production or consumption. Carbon-based flux maps are only balanced in terms of NADPH supply and biosynthetic demand if it is assumed that IDH generates NADPH rather than NADH [27,28,29,30].

An alternative approach to flux analysis, constraints-based metabolic modelling, takes explicit account of coenzyme balances, including the coenzyme requirements for both biosynthesis and maintenance [31]. Good fits have been obtained between model-predicted flux maps and carbon-based flux analysis, suggesting that the underlying assumptions about coenzyme specificity, and the contributions of NADPH and ATP to maintenance, are reasonable. However, this approach still relies on assumptions about the involvement of NADPH in particular processes, highlighting the need for a more direct assessment of coenzyme fluxes.

Deuterium-labelled substrates can be used to quantify the relative contribution of specific pathways to total NADPH production. A deuterated substrate is supplied to cells and deuterium is transferred onto the redox active hydride of NADPH via a specific pathway. Simultaneously, other NADPH-generating reactions will transfer unlabelled hydrogen onto the redox active hydride of NADPH. Therefore, the proportion of NADPH that has incorporated deuterium reflects the proportion that has been generated by the pathway being probed. By using different deuterated substrates, the contribution of different pathways to total NADPH production can be measured. This method has been successfully applied in mammalian cell cultures to quantify the relative NADPH production of various pathways such as the oxPPP using [1-^2^H]- or [3-^2^H]glucose [32,33,34], methylenetetrahydrofolate dehydrogenase using [2,3,3-^2^H]serine [32] and malic enzyme by using [4-^2^H]glucose [35]. However, analysis is complicated by the activity of flavin enzymes which are able to catalyse the loss of deuterium from the redox active hydride of NADPH to water via exchange involving flavin groups, requiring an additional correction to be applied [36,37]. This additional consideration means that genetic evidence is still important to support the conclusions of deuterium labelling studies [34].

The aim of the current work was to assess the practicality of using deuterium-labelled substrates to quantify the relative contribution of the oxPPP to NADPH production in heterotrophic *Arabidopsis* cell cultures, and to validate the method for future studies of other pathways using different deuterated substrates. To do this, LC-MS was used to measure deuterium incorporation into NADPH and other metabolites after supply of deuterated glucose or deuterated water (D_2_O). The effects of different amounts of substrate labelling and water exchange were simulated to establish their influence on the extent of deuterium incorporation into NADPH from labelled substrates. The effect of compartmentation was also simulated to quantify the effect disequilibrium between subcellular compartments can have on the accuracy of calculations based on whole cell extracts.

## 2. Results

### 2.1. Deuterium Labelling in Vitro

**Figure 1:**
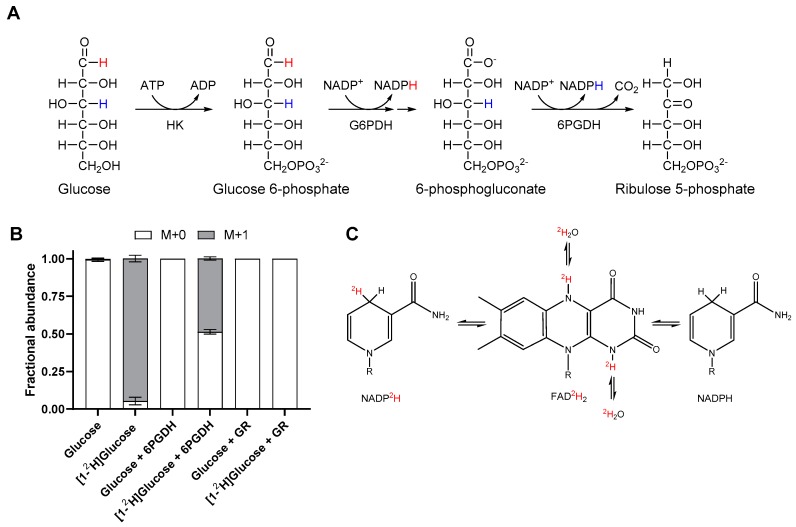
Validation of the labelling strategy for quantifying relative NADPH production via the oxPPP. (**A**) The route of deuterium incorporation from 1- (red) or 3- (blue) deuterated glucose into NADPH via glucose 6-phsophate dehydrogenase (G6PDH) or 6-phosphogluconate dehydrogenase (6PGDH). (**B**) Mass isotopologue distribution (MID) of NADPH after 4 h incubation with unlabelled or labelled substrate and in the presence or absence of glutathione reductase (GR) or 6PGDH. Samples were analysed using Zic-cHILIC chromatography and Xevo G2 XS MS. Values are the mean ± SD *n* = 3. (**C**) The route of hydrogen transfer between NADPH, FADH_2_ and water catalysed by flavin enzymes.

Redox reactions involving NAD(P)H transfer electrons as hydride ions and therefore isotopically labelled hydrogen can be used to directly follow these reactions. The oxPPP produces NADPH from glucose with hydrogen from carbon one or three being directly transferred to NADPH via glucose 6-phosphate dehydrogenase (G6PDH) and 6-phosphogluconate dehydrogenase (6PGDH), respectively (Figure 1A). To quantify the proportion of NADPH produced by the oxPPP, relative to all other sources, 1- or 3- deuterated glucose can be supplied to cells and the label incorporation into the redox active hydride of NADPH measured by LC-MS. To confirm this labelling strategy, [1-^2^H]glucose was combined with hexokinase (HK), G6PDH and 6PGDH and the mass isotopologue distribution (MID) of NADPH measured by LC-MS (Figure 1B). Supply of [1-^2^H]deuterated glucose to HK and G6PDH resulted in 94.7% ± 2.2% of NADPH incorporating a single deuterium atom. Upon the addition of 6PGDH, half of the NADPH produced was unlabelled as it had been produced by 6PGDH via transfer of unlabelled hydrogen from C3 of 6-phosphogluconate (6-PG). This confirmed that [1-^2^H]glucose could be used to quantify the proportion of NADPH produced by G6PDH and therefore the oxPPP.

In addition to transfer of deuterium onto NADPH from labelled substrates, the redox active hydride of NADPH can be exchanged with water via a reaction catalysed by flavin-containing enzymes (Figure 1C) [36,37]. This occurs as hydrogen is transferred from a non-labile C–H bond in NADPH to a more labile N–H bond in FADH_2_ [36,37]. This can result in loss of label from the redox active hydride of NADPH following labelling with deuterated substrates such as [1-^2^H]glucose, leading to an underestimation of the contribution of the oxPPP to total NADPH production. This effect was confirmed in vitro by the addition of glutathione reductase (GR), a flavin-containing enzyme, to a mixture of HK and G6PDH supplied with [1-^2^H]glucose. This resulted in the production of 100% unlabelled NADPH due to complete equilibration of the redox active hydride with water (Figure 1B). Incubation of GR with NADPH in 75% D_2_O in the absence of any other substrates also resulted in equilibration of NADPH with water (Figure S1), confirming that catalytic turnover of flavin enzymes was not necessary for the exchange to take place. The results of these in vitro experiments confirmed that [1-^2^H]glucose could be used to label NADPH via the oxPPP and highlighted the need to correct for the water exchange caused by flavin-containing enzymes.

### 2.2. Water Exchange in Vivo

To predict if the presence of NADPH-flavin enzymes could cause a significant exchange between NADPH and water in plants, the abundance of NADPH-flavin enzymes was compared in *A. thaliana* and *H. sapiens* proteomes available in PaxDB, an online quantitative proteomic database [38]. On average, *A. thaliana* proteomes contained significantly more NADPH-flavin enzymes than *H. sapiens (*Mann–Whitney test, *p* = 0.0002) (Figure S2), suggesting that flavin-enzyme-catalysed water exchange may be a greater issue in plants than in mammalian cells.

**Figure 2:**
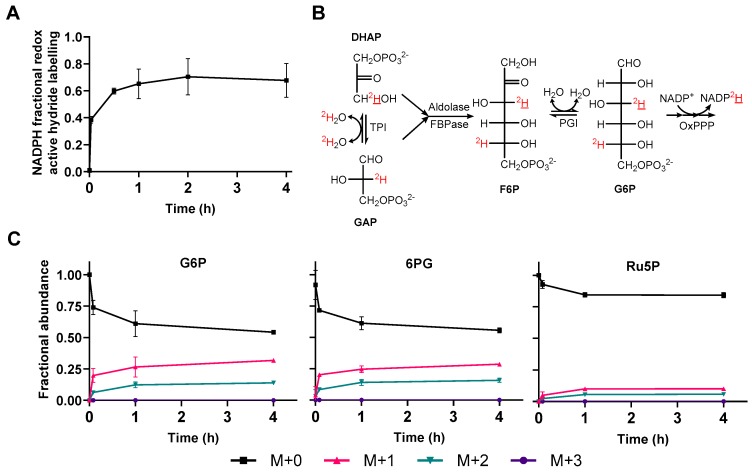
Deuterium labelling following transfer of *Arabidopsis* cells to 45% D_2_O. (**A**) NADPH redox active hydride labelling time course. Four-day-old heterotrophic *Arabidopsis* cells were transferred to media containing 45% D_2_O and extracted over 4 h before analysis by IPRP chromatography and Quattro Micro MS. Values are the mean ± SD, *n* = 3. (**B**) The route of deuterium (^2^H) incorporation into NADPH via triose phosphate isomerase (TPI) [41]. (**C**) Mass isotopologue distributions of oxPPP intermediates after incubation of *Arabidopsis* cell cultures on 45% D_2_O media for up to 4 h. Samples were analysed using IE chromatography and Q-Exactive MS and corrected for natural abundance using IsoCor [43]. Values are the mean ± SD, *n* = 3.

To quantify the hydrogen exchange between NADPH and water via the reaction with flavin-containing enzymes, heterotrophic *Arabidopsis* cells were incubated on media containing 45% D_2_O and the deuterium incorporation into the redox active hydride of NADPH quantified by LC-MS (Figure 2A). Deuterium was rapidly incorporated into the redox active hydride of NADPH and reached a steady state within 30 min (Figure 2A), consistent with data from mammalian cell cultures [36]. NADH also showed a similar pattern of deuterium incorporation (Figure S3). After 1 h, NADPH redox active hydride labelling reached an average of 68% ± 11%. Correcting for the 45% D_2_O in the media resulted in an exchange fraction of 150% ± 24%. An exchange fraction of >100% for a single hydrogen is unexpected but could be explained by exchange of an additional deuterium onto NADPH and not NADP^+^ at a site separate from the redox active hydride. In vivo, NADP^+^ and NADPH are rapidly interconverted so any deuterium incorporation at sites other than the redox active hydride is accounted for by deconvolution of the MIDs of NADP^+^ and NADPH (Equation (3)). Therefore, the additional exchange may have occurred after metabolism had been quenched and NADP^+^ and NADPH are no longer rapidly interconverted. Flavin enzymes not effectively inactivated after metabolite extraction could cause this additional exchange and therefore proteomic analysis was performed to identify if any proteins remained in the extract after organic solvent extraction (Figure S4). Across four independent cell extracts, 66 flavin-containing, NADPH binding, proteins were identified which may catalyse NADPH–water exchange. Despite this potential for an additional route of H/D exchange, it is clear that there is extensive exchange between the redox active hydride of NADPH and water, and that this may have implications for the application of deuterium labelling to *Arabidopsis* cell cultures.

In addition to direct deuterium incorporation into NADPH via flavin-enzyme-catalysed water exchange, deuterium could also be indirectly incorporated into the redox active hydride of NADPH from water via triose phosphates and the oxPPP (Figure 2B). Labile hydrogens in the active site of triose phosphate isomerase can exchange with those in water and be transferred onto triose phosphates [39,40,41]. Flux from triose phosphates back to hexose phosphates, as observed in heterotrophic tobacco cell cultures [42], can then lead to deuterium incorporation into positions within glucose 6-phosphate (G6P) that can be transferred onto NADPH via the oxPPP. This would result in the measured water exchange fraction being dependent on the flux from triose to hexose phosphates and the oxPPP, as well as the rate of flavin-enzyme-catalysed water exchange. Thus, quantifying the extent of labelling of NADPH from D_2_O may result in overestimation of the water exchange fraction and calculation of the oxPPP contribution to NADPH production. To determine if this occurred, the MIDs of oxPPP intermediates, G6P, 6PG and Ru5P were measured by LC-MS (Figure 2C). The proportion of M+1 and M+2 G6P and 6PG increased over time as deuterium was incorporated from D_2_O (Figure 2C). Less deuterium was incorporated into Ru5P suggesting that deuterium was transferred onto NADPH from C3 of 6PG via 6PGDH (Figure 1A) and therefore not present on Ru5P. This supports the hypothesis that the back flux from triose phosphates to G6P is sufficient to lead to significant label incorporation into NADPH. Ru5P still incorporates deuterium over time (Figure 2C), potentially via F6P and transketolases/transaldolases or from the oxPPP via G6P that may also be labelled at C5 (Figure 2B).

Incubation of cell cultures on D_2_O demonstrated rapid and extensive exchange of the redox active hydride of NADPH with water (Figure 2A), potentially preventing measurement of deuterium transfer onto the redox active hydride from deuterated glucose. In addition, the transfer of deuterium from water to NADPH via triose phosphates and the oxPPP causes overestimation of the water exchange fraction, preventing an accurate estimate of the contribution of the flavin-containing enzymes to water exchange.

### 2.3. [1-^2^H]Glucose Labelling in Vivo

**Figure 3:**
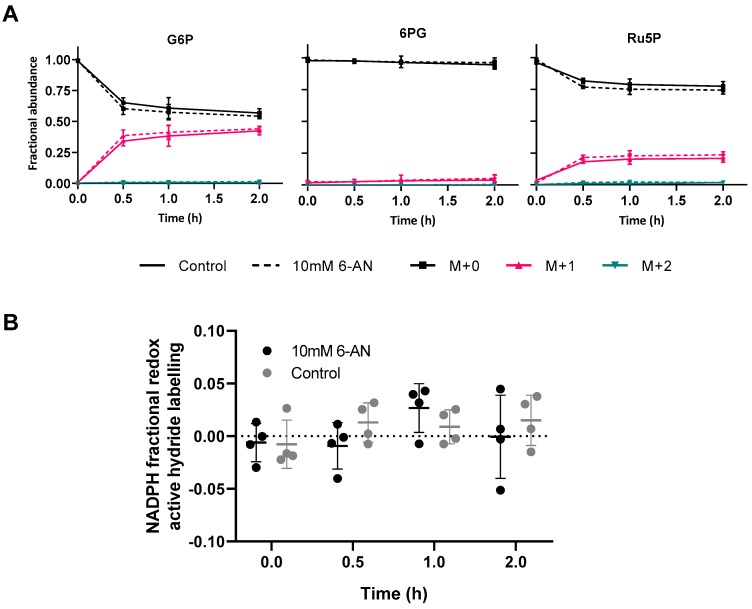
Deuterium labelling of metabolites following transfer of heterotrophic cells to 100% [1-^2^H]glucose media. (**A**) MIDs of metabolites after incubation of *Arabidopsis* cell cultures with (---) or without (–) 10 mM 6-AN for 20 h before transfer to 100% [1-^2^H]glucose media for up to 2 h. Samples were analysed using Zic-cHILIC chromatography, Xevo G2 XS MS and corrected for natural abundance using IsoCor [43]. Values are the mean ± SD, *n* = 4. (**B**) The effect of 6-AN on NADPH redox active hydride labelling calculated by deconvolution of NADP^+^ and NADPH MID. Cells were incubated with (•) or without(•) 10 mM 6-AN for 20 h before transfer to 100% [1-^2^H]glucose media and extracted over 2 h. Samples were analysed using Zic-cHILIC chromatography and Xevo G2 XS MS. Each point is a single biological replicate. Error bars are SD.

To establish if [1-^2^H] glucose can provide specific information about NADPH production from the oxPPP, despite the high rate of water exchange, heterotrophic *Arabidopsis* cells were transferred to media containing 100% [1-^2^H]glucose and the deuterium incorporation into NADPH and other metabolites was measured by LC-MS (Figure 3, Figures S5, S6). The effect of inhibiting the oxPPP using 6-aminonicotinamide (6-AN), an inhibitor of 6PGDH [44,45], was also investigated to determine if the expected NADPH labelling was dependent on the flux through the oxPPP.

Figure 3A shows the incorporation of deuterium over time into oxPPP intermediates. Deuterium is rapidly incorporated into the G6P pool, apparently reaching an isotopic steady state after ~1 h with 43.2 ± 2.7% becoming M+1 labelled. Deuterium was also incorporated into 6PG over time although to a much lesser extent than G6P. This suggested that deuterium was transferred onto NADPH from G6P and was therefore not present in 6PG. Ru5P showed greater deuterium incorporation than 6PG but less than G6P. Label can be incorporated into Ru5P from glycolytic intermediates and the non-oxidative PPP, similar to the result observed from D_2_O labelling (Figure 2C). Label incorporation into Ru5P can lead to downstream labelling of purines and subsequent labelling of the non-redox active hydrogens of NAD(P)(H) from de novo synthesis and salvage pathways. NAD is synthesized de novo from aspartate as well as being recycled from various break down products [46]. Incorporation of phosphoribosyl phosphonate (derived from R5P) by quinolinate phosphoribosyltransferase could lead to deuterium incorporation into NAD during de novo synthesis. Adenylation of nicotinate mononucleotide (NaMN) using ATP as a substrate could also lead to deuterium incorporation from both de novo synthesis and salvage pathways. Following transfer to [1-^2^H]glucose, deuterium was incorporated into ATP over time as well as NAD^+^ and NADH (Figure S5). This suggested that deuterium was incorporated into the non-redox hydrides of NADP(H) over 2 h.

NADPH can incorporate deuterium into one of two redox active hydrides from [1-^2^H]glucose via G6PDH (Figure 1A). The label incorporation specifically at the redox active hydride of NADPH was calculated by deconvolution of the NADP^+^ MID from the NADPH MID (Equation (3), Figure 3B). Although deuterium was incorporated into both NADPH and NADP^+^ (Figure S6), labelling of the redox active hydride was not significantly different from zero and did not change over time (one-way ANOVA *p* = 0.36) (Figure 3B). Unsurprisingly, inhibition of the oxPPP by addition of 6-AN had no effect on label incorporation, given that flux through the uninhibited oxPPP did not produce measurable redox active hydride labelling. NADH showed a similar lack of redox hydride labelling (Figure S7), although this was expected as G6PDH is specific to NADP^+^ [47]. The ability to measure small amounts of label incorporation depends on the precision of LC-MS analysis. The spectral accuracy of the LC-MS methods used in this study was determined by comparing the MID of unlabelled samples to the expected MID from natural abundance. For NADP(H) measured in this system, the absolute spectral error of the optimal method was ± 1.60% (Figure S8) meaning label incorporation lower than this cannot be reliably measured. The data show that there was no net accumulation of deuterium at the redox active hydride of NADPH when [1-^2^H]glucose was supplied, suggesting that the flux through the oxPPP was much smaller than the rate of flavin-enzyme-catalysed water exchange. Ultimately, the lack of detectable deuterium at the redox hydride of NADPH prevents measurement of the oxPPP contribution to NADPH production in heterotrophic *Arabidopsis* cells under the conditions tested here.

### 2.4. Simulating NADPH Redox Active Hydride Labelling

Deuterium incorporation into the redox active hydride of NADPH is dependent on the contribution of the pathway being measured to total NADPH production, the extent of substrate labelling, the water exchange fraction and the magnitude of any deuterium kinetic isotope effect (KIE) (Equations (1) and (2)). To assess the influence of these factors on the label incorporation into NADPH, different values for each of the parameters were simulated and the expected NADPH redox active hydride labelling calculated (Equations (1) and (2)).

**Figure 4:**
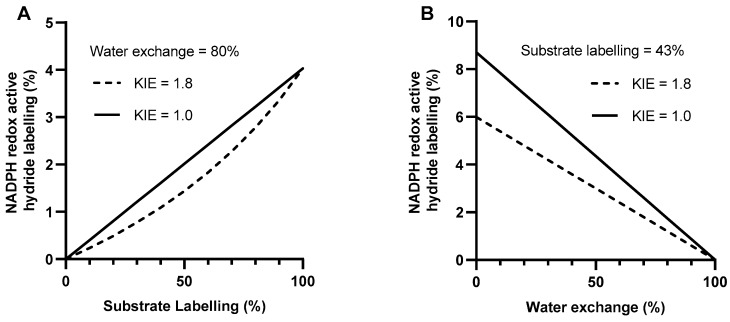
The simulated effect of substrate labelling (**A**) and water exchange fraction (**B**) on the NADPH redox active hydride labelling in the presence (---) or absence (–) of a deuterium kinetic isotope effect (KIE) calculated using Equations (1) and (2) (see Section 5.4). The oxPPP contribution to NADPH production was fixed at 40%. Water exchange was fixed at 80% whilst substrate labelling was varied (**A**) Substrate labelling was fixed at 43% whilst water exchange was varied (**B**) Calculations were performed using linear equations in MATLAB (see Supplementary Materials for link to code).

Figure 4 shows the effect of different water exchange and substrate labelling fractions on the label incorporation into NADPH, assuming 40% of total cellular NADPH is produced by the oxPPP [29]. As substrate labelling increases, the NADPH labelling increases (Figure 4A) and as water exchange increases, NADPH labelling decreases (Figure 4B). The presence of a kinetic isotope effect (KIE) can also decrease the observed NADPH labelling as hydrogen can be preferentially transferred onto NADPH over deuterium. These data highlight the fact that deuterium incorporation into NADPH can be significantly decreased by high water exchange and low substrate labelling as well as the deuterium kinetic isotope effect, which may differ depending on the enzyme or pathway being analysed.

### 2.5. The Effect of Subcellular Compartmentation

**Figure 5:**
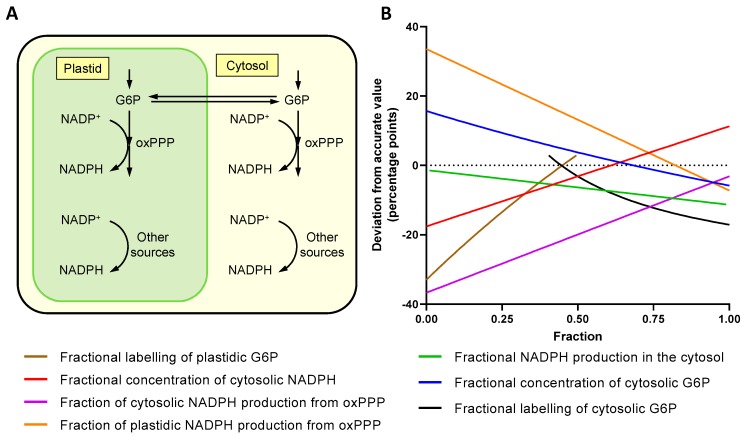
The effect of compartmentation on the measurement of the oxPPP contribution to total NADPH production. (**A**) Two compartment model simulating duplicated oxPPP in the cytosol and plastids. (**B**) The deviation of the experimentally measured value for a whole cell extract from the actual value for the oxPPP contribution to total cellular NADPH production. Each parameter was varied between 0 and 1 whilst all other parameters were fixed at the following values: fractional amount of cytosolic G6P, 0.85 [49]; fractional amount of cytosolic NADPH, 0.5 [50,51]; fractional NADPH production in the cytosol, 0.18 [29]; fraction of cytosolic NADPH production from oxPPP, 1.0 [29]; fraction of plastidic NADPH production from oxPPP, 0.9; fractional labelling of cytosolic G6P, 0.5; and fractional labelling of plastidic G6P, 0.4. The model was constructed using linear equations in MATLAB (see Supplementary Materials for link to code).

Subcellular compartmentation can affect the interpretation of labelling data from whole cell extracts. Unlike mammalian cells, the oxPPP is present in both the cytosol and plastids of *Arabidopsis* [27,48] with the potential for different fluxes in each compartment. Depending on the extent of equilibration of substrates and isotopic label between compartments, analysis of whole cell extracts could produce an incorrect estimate of the oxPPP contribution to NADPH production. To identify how subcellular compartmentation could affect the accuracy of the experimental method, a linear model was constructed (Figure 5A), and each parameter varied in turn whilst all others remained fixed (Figure 5B).

For example, Figure 5B shows that if labelling of G6P in the cytosol and plastid does not reach isotopic equilibrium then the measured value of the total oxPPP contribution to NADPH production could be underestimated by up to 30 percentage points (pp). Other parameters also cause deviations (up to 37 pp) depending on how much they differ between compartments. These data highlight the fact that compartmentation can cause variable, non-proportional errors when making calculations based on whole cell extracts, with disequilibrium between cytosolic and plastidic G6P labelling potentially having one of the largest effects.

## 3. Discussion

Deuterium was not detected at the redox active hydride of NADPH after feeding cells with 100% [1-^2^H]glucose (Figure 3B). The lack of redox hydride labelling may be explained by high flavin-enzyme-catalysed water exchange and low substrate labelling, with further complications from triose/hexose phosphate exchange and compartmentation, ultimately confounding the proposed method for measuring the oxPPP contribution to NADPH production in heterotrophic *Arabidopsis* cell cultures.

### 3.1. Flavin-Enzyme-Catalysed Water Exchange Abolishes Detectable Labelling from [1-^2^H]glucose

Flavin enzymes catalyse exchange between NADPH redox active hydride and water via a half reaction that does not require catalytic turnover [36]. This was confirmed in vitro with glutathione reductase and NADPH in the absence of glutathione, with NADPH completely equilibrating with water (Figure 1B, Figure S1). In heterotrophic *Arabidopsis* cell cultures, NADPH rapidly incorporated deuterium from D_2_O with a water exchange fraction >100% (Figure 2A). In contrast, mammalian cell cultures show lower water exchange fractions, from 40% to 70% depending on the cell line [36]. The higher water-exchange fraction in plants is likely due to a greater abundance of flavin enzymes in *A*. *thaliana* compared to *H. sapiens* cell lines, as shown by quantitative proteomic data from PaxDB [38] (Figure S2). However, the magnitude of the water exchange fraction is ultimately dependent on the relative rates of flavin-enzyme-catalysed water exchange and all other NADPH producing reactions. In some organisms, cell lines, or under certain conditions, the flux through NADPH producing reactions may be slow relative to the flavin-enzyme-catalysed water exchange, preventing detectable accumulation of deuterium at the redox active hydride of NADPH following supply of deuterated substrates, as is the case in heterotrophic *Arabidopsis* cell cultures (Figure 3B). Deuterium was still incorporated into NADP^+^ and NADPH (Figure S6) at sites other than the redox active hydride most likely from de novo synthesis and salvage pathways of NAD metabolism.

### 3.2. Low Substrate Labelling Decreases Maximum Possible Labelling Signal

As well as high water exchange, low substrate labelling may also decrease the extent of deuterium incorporation into NADPH below detectable levels (Figure 4). In this study, labelling of G6P, the immediate precursor for label transfer to NADPH, reached only 43.2 ± 2.7% M+1 after 2 h (Figure 3A). This is significantly less than the substrate labelling reported for mammalian cell cultures which ranges from 60% to 100% depending on the cell line and glucose substrate used [32,33,36]. Deuterium from [1-^2^H]glucose can be lost due to the reversible interconversion catalysed by a combination of phosphoglucose and phosphomannose isomerases [52]. Hexose to triose phosphate recycling could also transfer deuterium from carbon one to carbon six of G6P [42]. Use of [3-^2^H]glucose may increase the labelling of G6P by avoiding these losses, although triose-hexose phosphate exchange could still cause loss of deuterium from C3 by exchange with water. In H1299 human cells, use of [3-^2^H]glucose increased G6P labelling from 68% to 90% [33] although use of [3-^2^H]glucose did not significantly affect G6P labelling in iBMK cells [32]. The relatively low level of substrate labelling achieved in *Arabidopsis* cells limits the maximum possible labelling in the redox active hydride of NADPH to 1.9%, assuming a KIE of 1.8 [53], an oxPPP contribution of 40% and a water exchange fraction of 80% (Figure 4B). This approaches the analytical precision of the LC-MS methodology (Figure S8), suggesting that even if the water exchange fraction were lower, accurate measurement of NADPH labelling would remain a challenge.

### 3.3. Metabolic Network Structure Prevents Accurate Measurement

Whilst higher rates of NADPH production under certain conditions or in certain cell lines may result in a detectable level of deuterium in the redox active hydride of NADPH, the structure of the plant metabolic network compromises quantitative analysis due to potential overestimation of the water exchange fraction. Cells grown on media containing D_2_O can incorporate deuterium into metabolites via any reaction where hydrogen is exchangeable with water. A prominent route in photosynthetic cells is the reduction of NADP^+^ by ferredoxin-NADP^+^ reductase, which specifically uses hydrogen from water to produce NADPH. This problem is circumvented in the current study by the use of heterotrophic plant cells and the extraction of metabolites under green light. However, even in heterotrophic tissues, many enzymes in which reaction intermediates possess labile hydrogens such as isomerases, hydratases and aminotransferases, can also incorporate deuterium from water into various metabolites. One such route is via triose phosphate isomerase where deuterium from D_2_O can become incorporated onto C3 of DHAP and is retained on C3 of hexose phosphates [41] (Figure 2B,C). *Arabidopsis* cells cultured on 45% D_2_O show equivalent labelling patterns in G6P and 6PG but significantly less label incorporation into Ru5P (Figure 2C), suggesting that deuterium is at C3 of 6PG and specifically transferred to NADPH via 6PGDH. This resulted in NADPH labelling from D_2_O being dependent on both flavin-enzyme-catalysed water exchange and triose-phosphate recycling combined with oxPPP flux. Therefore, the water exchange fraction cannot be accurately determined in this system. This is similar to an issue in a mammalian system discussed by Zhang et al., [36] in which additional deuterium incorporation into malate from D_2_O via fumarase may lead to labelling of NADPH via malic enzyme (ME) and therefore cause overestimation of the water exchange fraction in cells where ME makes a significant contribution to NADPH production.

Finally, the compartmentation of plant metabolism compromises the accuracy of flux determination from deuterium labelling. In contrast to mammalian cells, the oxPPP in plants is duplicated in the cytosol and plastids [27,48] and the extent of equilibration between compartments can affect measurements based on whole cell extracts (Figure 5). The simple model used in this study was based on just two compartments, whilst in reality, contributions to NADPH supply from the mitochondria and peroxisomes could also affect results, further decreasing the quantitative accuracy of measurements from whole cell extracts. Subcellular deuterium incorporation could potentially be resolved for some metabolites such as G6P where UDP- and ADP-glucose can be used to report on cytosolic and plastidic G6P labelling respectively [54]. Similar labelling patterns were measured for G6P (Figure 3A) and UDP-glucose (Figure S5), following supply of [1-^2^H]glucose, suggesting that the G6P from whole cell extracts is representative of the cytosolic pool. This may be due to the relatively high cytosolic concentration of G6P [49,55,56], or rapid equilibration between cytosolic and plastidic pools of G6P. However, evidence from ^13^C MFA of heterotrophic *Arabidopsis* cell cultures shows that cytosolic and plastidic pools of G6P are not in isotopic equilibrium [27]. Disequilibrium between hexose phosphate pools in different compartments could be quantified by comparing label incorporation in to UDP-glucose and ADP-glucose but unfortunately the concentration of ADP-glucose was too low for reliable analysis in this study.

## 4. Conclusions

Whilst the methods explored here have previously been exploited to study redox metabolism in mammalian cell cultures [32,33,34,35,36], several factors confound the use of the same approach in plant cells. The plant metabolic network is markedly different from that of mammals, being more compartmented and interconnected. These differences affect the interpretation of deuterium labelling experiments, ultimately preventing the quantitative measurement of the oxPPP contribution to total NADPH production. Low substrate labelling and high water-exchange combine to produce a level of NADPH labelling that is close to, or below, the limit of analytical detection. Even if analytical precision were improved, the accuracy of the estimates is likely to be compromised due to overestimation of the NADPH–water exchange fraction and potential errors introduced by compartmentation. Future work will rely on alternative approaches for resolving coenzyme fluxes, potentially using in vitro enzyme specificities to put bounds on NADPH production rates from different sources [57]. Overall, the findings presented here provide a framework for establishing deuterium labelling strategies to quantify redox fluxes in organisms other than mammalian cells. Although it is disappointing that the method is not tenable in heterotrophic *Arabidopsis* cell cultures, these tools will hopefully still provide insight into other biological systems in the future.

## 5. Materials and Methods

### 5.1. Arabidopsis Cell Culture

Cell suspensions of *Arabidopsis thaliana* (ecotype Landsberg *erecta*) were cultured in MS medium containing 30 g/L glucose, 0.5 mg/L 1-naphthaleneacetic acid, and 0.05 mg/L kinetin as described by Williams et al. [58]. Cultures were grown in sterile 250 mL Erlenmeyer flasks, sealed with a double layer of aluminium foil, on an orbital shaker running at 120 rpm in complete darkness at 22 °C. The cell line was maintained by weekly subculturing 15 mL of cell culture into 100 mL of fresh medium.

A method for growing cell cultures on easily transferable filter paper disks was modified from Horsch et al. [59] and Scholten et al. [60]. Filter paper cultures of *A. thaliana* were maintained on 42.4 mm diameter Whatman No. 1 filter papers in 5 cm diameter Petri dishes containing 5 mL MS agar (0.5% *w/v*) with 30 g/L glucose, 0.5 mg/L 1-naphthaleneacetic acid, and 0.05 mg/L kinetin at 22 °C. Dishes were sealed with a layer of Micropore (3M) tape and grown in complete darkness. An amount of 2 mL of 4-d-old liquid cultures was collected on filter papers using a Buchner funnel and concentrated by weak vacuum before the cells and filter paper were transferred directly to agar plates. Filter paper cultures were then grown for 4 d (unless otherwise specified) before use in isotopic labelling experiments.

### 5.2. Metabolite Extraction

Under green light, cells were scraped from filter papers into a 2 mL screw-cap microfuge tube, weighed, and an appropriate volume of ice-cold extraction solvent (1:1 MeOH:AcN) was added in a ratio of cell fresh weight (mg) to extraction solvent (μL) of 1:1.5 (unless otherwise stated). The tube was then vortexed for 5 s and submerged in liquid nitrogen before being centrifuged for 20 min at 20,000× *g* at 4 °C. The supernatant was then filtered through 10 KDa MWCO filters (Amicon) before being transferred to Waters total recovery LC-MS vials and either analysed immediately or stored at −80 °C.

### 5.3. Liquid Chromatography Mass Spectrometry

Method 1—Metabolite extracts were analysed using a Thermo Scientific ICS-5000+ ion chromatography system coupled directly to a Q-Exactive Hybrid Quadrupole-Orbitrap mass spectrometer with a HESI II electrospray ionization source (Thermo Scientific, San Jose, CA, USA). The ICS-5000+ HPLC system incorporated an electrolytic anion generator (KOH) which was programmed to produce a 5–100 mM OH^−^ gradient over 37 min to facilitate ion chromatography. An inline electrolytic suppressor removed hydroxide ions and cations from the post-column eluent prior to MS analysis (Thermo Scientific Dionex AERS 500). A 10 μL partial loop injection was used for all analyses and the chromatographic separation was performed using a Thermo Scientific Dionex IonPac AS11-HC 2 × 250 mm, 4 μm particle size column with a Dionex Ionpac AG11-HC 4 μm 2 × 50 guard column inline. The flow rate was 0.25 mL/min. The total run time was 37 min and the hydroxide ion gradient was: 0 min, 0 mM; 1 min, 0 mM; 15 min, 60 mM; 25 min, 100 mM; 30 min, 100 mM; 30.1 min, 0 mM; 37 min, 0 mM. Analysis was performed in negative ion mode using a scan range from 60 to 900 and resolution set to 70,000. The tune file source parameters were: Sheath gas flow 60 mL/min; auxiliary gas flow 20 mL/min; spray voltage 3.6 V; capillary temperature 320 °C; S-lens RF value 70; heater temperature 450 °C. Automatic gain control target was set to 1 × 10^6^ and the maximum injection time value was 250 ms. The column temperature was maintained at 30 °C.

Method 2—An ion-pairing reverse phase chromatography method was modified from that reported by Buescher et al. [61]. Compounds were separated using a silica based reverse phase C18 column (Waters Cortecs T3, 1.6 μm, 2.1 × 100 mm). The mobile phase solvent A was 10 mM tributylamine, 10 mM acetic acid, 2 mM acetyl acetone, 5% (*v/v*) methanol all in Milli-Q water and solvent B was propan-2-ol. The chromatographic gradient was: 0 min, 100% A, 0.35 mL/min; 3 min, 92% A, 0.35 mL/min; 5 min, 92% A. 0.35 mL/min; 10 min 87% A, 0.35 mL/min; 10.5 min, 87% A, 0.15 mL/min; 12 min 20% A, 0.15 mL/min; 18 min, 20% A, 0.12 mL/min; 21 min, 100% A, 0.15 mL/min; 22 min, 100% A, 0.35 mL/min. The column temperature was maintained at 40 °C. MS analysis was performed using a Quattro-Micro triple quad MS (Waters, Milford, MA, USA) in negative ion mode using single ion monitoring for each compound and isotopologue. The source parameters were: desolvation gas flow 190 L/hr; cone gas flow 20 L/h; capillary voltage 2.7 V; source temperature 150 °C; desolvation temperature 390 °C; cone voltage 20 V.

Method 3—Compounds were separated using a Zic-cHILIC (3 μm, 100 Å, 150 × 2.1 mm) column (Merck, Kenilworth, NJ, USA). The mobile phase solvent A was 20 mM ammonium acetate in water and solvent B was 100% acetonitrile (AcN). The flow rate was 0.4 mL/min and the gradient was: 0 min, 30% A; 1 min, 30% A; 10 min, 40% A; 10.5 min, 60% A; 15 min, 60% A; 15.5 min, 30% A; 19 min 30% A. The injection volume was 2 µL using partial loop injection in a 10 µL loop. The column temperature was maintained at 40 °C. MS analysis was performed using a Xevo-G2-XS (Waters, Milford, MA, USA) in negative mode, scan range from 50 to 900. Source parameters were: desolvation gas flow 750 L/h; cone gas flow 50 L/hr; capillary voltage 2.5 V; source temperature 150 °C; desolvation temperature 300 °C; cone voltage 40 V; source offset 80 V.

### 5.4. Simulating NADPH Redox Active Hydride Labelling

A MATLAB script was written to compute the effect of changing each parameter on the measured NADPH redox active hydride labelling. The script is based on a correction factor for the deuterium kinetic isotope effect (Equation (1)) and the calculation of the relative contribution of a pathway to total NADPH production (Equation (2)) based on [32,36] (see Supplementary Materials for link to code).

(1) $$C_{\mathrm{KIE}}=\;\frac{V_{H}}{V_{D}}+x\left(1-\;\frac{V_{H}}{V_{D}}\right)$$

**Equation (1):** Correction factor for deuterium kinetic isotope effect. V_H_/V_D_ is the preference of the enzyme for hydrogen over deuterium. x is the substrate labelling fraction.

(2) FractionNADPH from oxPPP=2 × ([H 2]NADPHTotal NADPH)×([H 2]G6PTotal G6P)−1×(1−Hexchange)−1×CKIE

**Equation (2):** Calculation of the contribution of the oxPPP to total NADPH production. Terms in brackets are the fractional labelling of NADPH redox active hydride, fractional labelling of G6P at C1 and the correction factor for water exchange of NADPH redox active hydride. C_KIE_ is the correction factor for the deuterium kinetic isotope effect.

### 5.5. Redox Active Hydride Labelling

Calculation of NADPH redox active hydride labelling (Equation (3)) was adapted from Fan et al., [32]. The equation was implemented in MATLAB and code is available online (see Supplementary Materials for link to code).

Observed MID vector of NADP^+^ from a labelled sample where $a_{N}$ is the proportion of isotopologues containing N heavy isotopes:

$$NADP^{+}{}_{labelled}=\left[\;\begin{array}{c} M+0\\ M+1\\ M+2 \end{array}\right]\;\equiv \left[\;\begin{array}{c} a_{0}\\ a_{1}\\ a_{2} \end{array}\right]$$

The observed MID vector of NADPH from the same labelled sample as $NADP^{+}{}_{labelled}$ described in terms of $NADP^{+}{}_{labelled}$ and the parameter x:

(3) $$NADPH_{labelled}\;=\left[\;\begin{array}{c} M+0\\ M+1\\ M+2 \end{array}\right]\;=\left[\;\begin{array}{c} a_{0}\left(1-x\right)\\ a_{1}\left(1-x\right)+\;a_{0}x\;\\ a_{2}\left(1-x\right)+\;a_{1}x \end{array}\right]\;\cdot \;\frac{1}{1-\left(a_{2}x\right)}$$

**Equation(3):** The relationship between the observed MID of NADPH and NADP^+^ from the same sample. M+N is the observed proportion of isotopomers containing N heavy isotopes. x is the fractional enrichment of the redox active hydride of NADPH.

### 5.6. In Vitro oxPPP Labelling

The reaction mix contained 20 mM NH_4_HCO_3_ (pH 8.0), 5 mM MgCl_2_, 0.5 mM NADP^+^, 0.5 mM ATP, 0.2 mM glucose, 0.5 U/mL hexokinase (*S. cerevisiae*), 0.5 U/mL glucose 6-phosphate dehydrogenase (*L. mesenteroides*), 0.5 U/mL 6-phosphogluconate dehydrogenase (*S. cerevisiae*) and 2.5 U/mL glutathione reductase (*S. cerevisiae*). Aliquots (200 μL) of reaction mix were incubated at room temperature for 4 h in a 96-well plate reader. Absorbance at 340 nm was monitored to ensure the reaction was complete. An amount of 200 μL of reaction mixture was then added to 800 μL of 4 °C quenching buffer (1:1 MeOH:AcN) centrifuged for 15 min at 20,000× *g* at 4 °C and the supernatant stored at −80 °C before analysis by LC-MS.

### 5.7. In Vitro NADPH–Water Exchange by Glutathione Reductase

An amount of 0.7 mM NADPH was incubated at room temperature in an aqueous reaction mixture containing 2.5 mM Tris-Cl (pH 7.6), 5 mM NaCl, 8.5 U/mL glutathione reductase and 75% D_2_O. After 40 min, 200 μL of reaction mixture was added to 800 μL of 4 °C quenching buffer (1:1 MeOH:AcN with or without 0.625% (*v/v*) formic acid) and incubated on dry ice for 20 min. Samples were then centrifuged for 15 min at 20,000× *g* at 4 °C and the supernatant placed in autosampler vials for immediate analysis by LC-MS.

### 5.8. In Vivo D_2_O Labelling

Four-day-old heterotrophic *Arabidopsis* filter paper cultures were washed with liquid MS media containing 30 g/L glucose, 0.5 mg/L 1-naphthaleneacetic acid, and 0.05 mg/L kinetin and 45% D_2_O. Cells were then either immediately extracted or transferred to MS agar plates containing 45% D_2_O and extracted at 0.5, 1, 2 and 4 h post transfer to deuterated media.

### 5.9. In Vivo [1-^2^H]glucose Labelling and 6-AN Treatment

Four-day-old heterotrophic *Arabidopsis* filter paper cultures were transferred to MS agar plates with or without 10 mM 6-AN and incubated for 20 h before being transferred to MS agar plates containing 100% [1-^2^H]glucose (30 g/L) (98% isotopic purity; Cambridge Isotope Laboratories, Andover, MA, USA) and 10 mM 6-AN. Cells were then incubated in the dark and metabolites extracted at 0.5, 1 and 2 h post transfer to deuterated media.

### 5.10. Proteomic Analysis of Cell Extract

Four-day-old heterotrophic *Arabidopsis* filter paper cultures were extracted as described in 5.2 and the MWCO filter concentrate was analysed via tryptic digest proteomics. Samples were reduced with 10 mM dithiothreitol (DTT) for 40 min at 56 °C before alkylation in 20 mM iodoacetamide (IAA) for 30 min in the dark at room temperature. Samples were reduced again with 3 uL of 85 mM DTT for 10 min in the dark at room temperature to eliminate excess IAA. Samples were then precipitated with six volumes of ice-cold acetone and incubated overnight at −20 °C before centrifugation at 15,000× *g* for 10 min at 4 °C. The pellet was dried in air for no more than 5 min before resuspension in 50 mM ammonium bicarbonate. Trypsin was then added in a 1:50 (*w/w*) ratio of trypsin:sample protein and incubated overnight at 37 °C. A second trypsin digestion was performed with a 1:100 (*w/w*) ratio in 80% acetonitrile and incubated at 37 °C for three hours. The reaction was stopped by addition of 5% formic acid and the sample dried using a vacuum centrifuge. The peptides were resolubilised in 15 µL of 0.1% formic acid.

Resulting tryptic peptides were analysed on a NanoAcquity-UPLC system (Waters) connected to an Orbitrap Elite mass spectrometer (Thermo Fischer Scientific) possessing an EASY-Spray nano-electrospray ion source (Thermo Fischer Scientific). The peptides were trapped on an in-house packed guard column (75 μm i.d. × 20 mm, Acclaim PepMap C18, 3 μm, 100 Å) using solvent A (0.1% formic acid in water) at a pressure of 140 bar. The peptides were separated on an EASY-spray Acclaim PepMap® analytical column (75 μm i.d. × 50 mm, RSLC C18, 3 μm, 100 Å) using a linear gradient (length: 100 min, 3% to 60% solvent B (0.1% formic acid in acetonitrile), flow rate: 300 nL/min). The separated peptides were electrosprayed directly into the mass spectrometer operating in a data-dependent mode using a CID based method. Full scan MS spectra (scan range 350–1500 m/z, resolution 120,000, AGC target 1 × 10^6^, maximum injection time 250 ms) and subsequent CID MS/MS spectra (AGC target 5 × 10^4^, maximum injection time 100 ms) of the 10 most intense peaks were acquired in the Ion Trap. CID fragmentation was performed at 35% of normalized collision energy and the signal intensity threshold was kept at 500 counts. The CID method used performs beam-type CID fragmentation of the peptides.

Data analysis was performed with Peaks 8.5 (Bioinformatics Solutions Inc., Waterloo, ON, Canada) The raw MS file was searched against the respective protein sequence. Trypsin with a maximum number of 3 missed cleavages and one unspecific end was selected as the protease. Carbamidomethylation (cysteine), oxidation (methionine), deamination (asparagine, glutamine) were set as variable modifications. Precursor mass tolerance was set as 15 ppm and fragment mass tolerance for CID was set to 0.8 Da.

### 5.11. PaxDB Quantitative Proteome Analysis

Data from PaxDB [38] was associated with data from Uniprot [62] to identify the relative abundance of proteins that contain NADP(H) and FAD binding sites. PaxDB data for 37 *A. thaliana* and 40 *H. sapiens* cell line proteomes were downloaded from PaxDB as .csv files. PaxDB identifiers were matched to Uniprot accession numbers which were then used to download information about the proteins. Proteins which were associated with both “FAD” and “NADP” keywords were identified as being flavin enzymes, potentially capable of catalysing hydrogen exchange between NADPH and water. The relative abundance of these proteins was summed to give a total abundance of NADP-flavin enzymes in each proteome.

### 5.12. Compartmentation Simulation

A linear model was created in MATLAB consisting of seven variable parameters: fractional amount of cytosolic G6P, 0.85 [49]; fractional amount of cytosolic NADPH, 0.5 [50,51]; fractional NADPH production in the cytosol, 0.18 [29]; fraction of cytosolic NADPH production from oxPPP, 1.0 [29]; fraction of plastidic NADPH production from oxPPP, 0.9; fractional labelling of cytosolic G6P, 0.5; and fractional labelling of plastidic G6P, 0.4. Each parameter was varied between 0 and 1 whilst all other parameters were fixed. Note that plastidic G6P labelling fractions greater than cytosolic G6P labelling were omitted, as it was assumed all plastidic G6P is derived from cytosolic G6P. See Supplementary Materials for link to code.

## Supplementary Materials

The following are available online at https://www.mdpi.com/2218-1989/9/10/205/s1, Figure S1: In vitro deuterium incorporation into NADPH from D_2_O catalysed by glutathione reductase, Figure S2: Abundance of NADP-flavin enzymes in *A. thaliana* compared to *H. sapiens*, Figure S3: NADH redox active hydride labelling following supply of D_2_O, Figure S4: NADP-flavin enzymes present in cell extracts, Figure S5: MIDs of metabolites following transfer to 100% [1-^2^H]glucose media, Figure S6: NADP(H) MIDs following transfer to 100% [1-^2^H]glucose media, Figure S7: NADH redox active hydride labelling following transfer to 100% [1-^2^H]glucose media, Figure S8: Spectral accuracy of NADP(H) MID measurement. Code used in this work is available online at www.github.com/tednsmith/Deuterium-Labelling-Arabidopsis.
