# Supplementary material for: Limitations of Deuterium-Labelled Substrates for Quantifying NADPH Metabolism in Heterotrophic Arabidopsis Cell Cultures
Source: Metabolites. 2019 Sep 28;9(10):205. doi: 10.3390/metabo9100205 (PMC6835633; doi:10.3390/metabo9100205)
Supplement: Supplementary file 1 [file metabolites-09-00205-s001.pdf]

Supplementary data

		Page
**Figure S1**	In vitro deuterium incorporation into NADPH from D_2O catalysed by glutathione reductase	**2**
**Figure S2**	Abundance of NADP-flavin enzymes in *A. thaliana* compared to *H. sapiens*	**3**
**Figure S3**	NADH redox active hydride labelling following supply of D_2O	**3**
**Figure S4**	NADP-flavin enzymes present in cell extracts	**4**
**Figure S5**	MIDs of metabolites following transfer to 100% [1-^{2}H]glucose media	**5**
**Figure S6**	NADP(H) MIDs following transfer to 100% [1-^{2}H]glucose media	**6**
**Figure S7**	NADH redox active hydride labelling following transfer to 100% [1-^{2}H]glucose media.	**7**
**Figure S8**	Spectral accuracy of NADP(H) MID measurement	**8**

To confirm flavin enzymes catalyse exchange between NADPH redox hydride and water NADPH was combined with glutathione reductase in 75% D_2O and incubated for 40 min (Figure S1). The effect of adding formic acid (FA) to the quenching solvent was also measured as it was shown that formic acid can improve extraction efficiency of NADPH [1]. Glutathione reductase catalysed H/D exchange between NADPH and water (Figure S1), consistent with published data [2]. However, formic acid also caused deuterium incorporation from D_2O in the absence of GR which is inconsistent with published data [2]. M+2 ions were present when NADPH was incubated with GR and then quenched with solvent containing formic acid. This showed that acid catalysed H/D exchange was at a site distinct from that of GR catalysed exchange which is presumed to be at the redox active hydride [2]. It is possible that the acid catalysed exchange was also at the redox active hydride of NADPH but is not stereospecific for either of the two redox hydrides of NADPH. Because of the potential for additional exchange, formic acid was omitted from the extraction solvent for subsequent extraction of cell cultures.

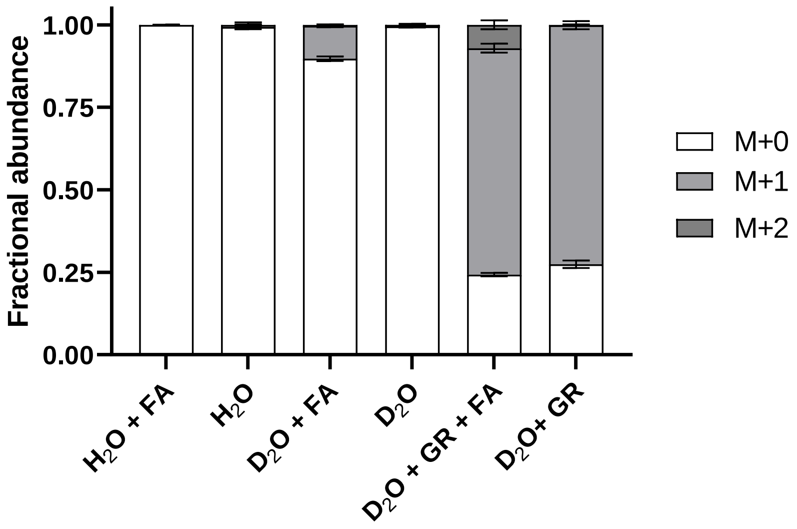

Figure S1. In vitro deuterium incorporation into NADPH from D_2O catalysed by glutathione reductase. 0.7mM NADPH was incubated with 8.5 Unit/mL GR in 2.5mM Tris (pH 7.6) with either 100% H_2O or 75% D_2O for 40 min. The reaction was quenched by adding 800 µl of 4 °C quenching buffer (1:1 MeOH:AcN with or without 0.625% formic acid (FA) to 200 µl of reaction mix. MIDs are corrected for natural abundance of NADPH using IsoCor. Values are mean ± SD, n = 3.

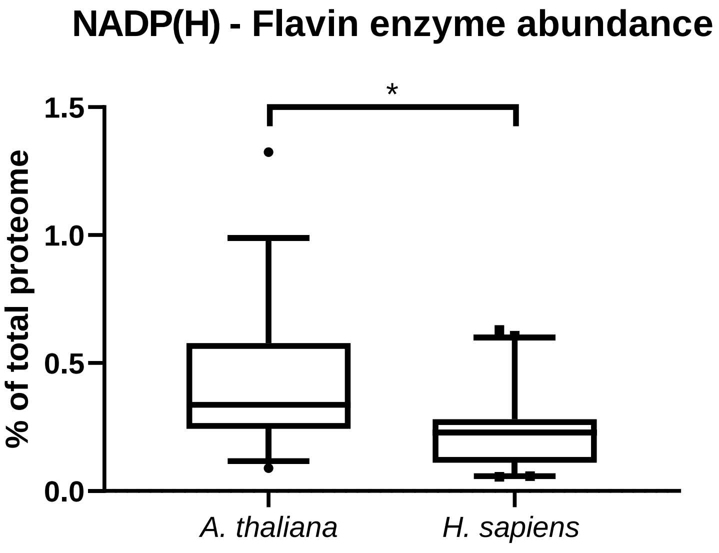

Figure S2. Abundance of NADP(H) flavin enzymes from PaxDB [3]. NADP(H) flavin enzymes were categorized as having FAD and NADP(H) binding sites. Data points are from 37 *A. thaliana* proteomes and 40 *H. sapiens* proteomes. Populations are significantly different (Mann-Whitney test, p = 0.0002)

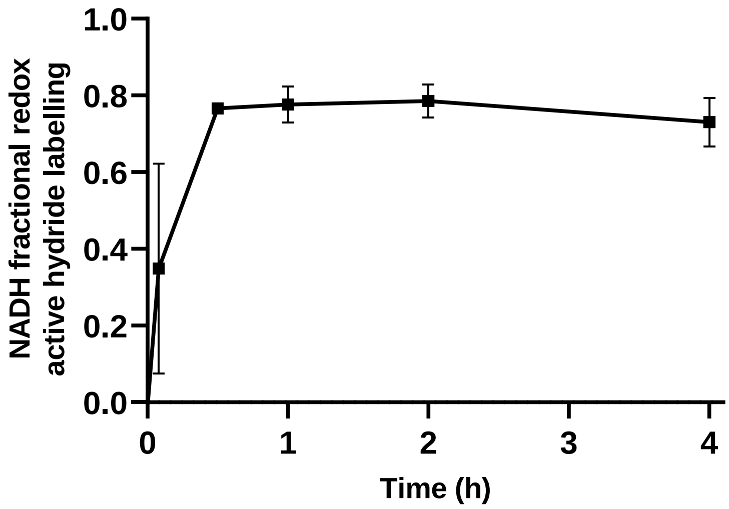

Figure S3 NADH redox active hydride labelling following transfer to D_2O media. 4-d-old heterotrophic *Arabidopsis* cells were transferred to media containing 45% D_2O and extracted over 4 h before analysis by IPRP chromatography and Quattro Micro MS. Values are the mean ± SD, n = 3.

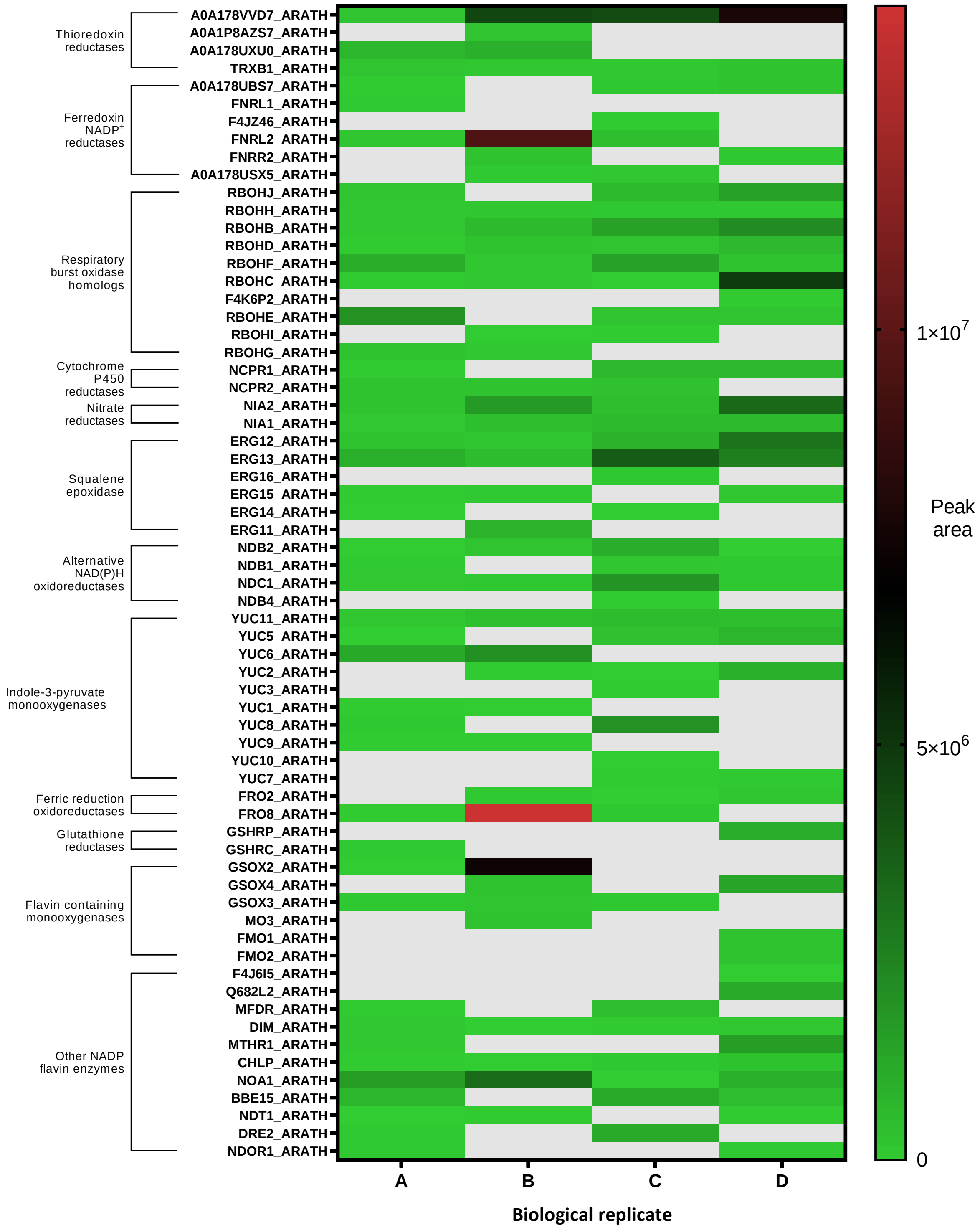

Figure S4. Heatmap showing the peak area of NADP-flavin enzymes identified in heterotrophic *Arabidopsis* cell extracts prior to MWCO filtration. Across the four samples analysed 66 unique proteins were identified which may catalyse NADPH solvent exchange. There was a significant amount of variability with some proteins only identified in a single sample. This highlights protein precipitation as a potential source of variability in sample preparation and metabolite extraction.

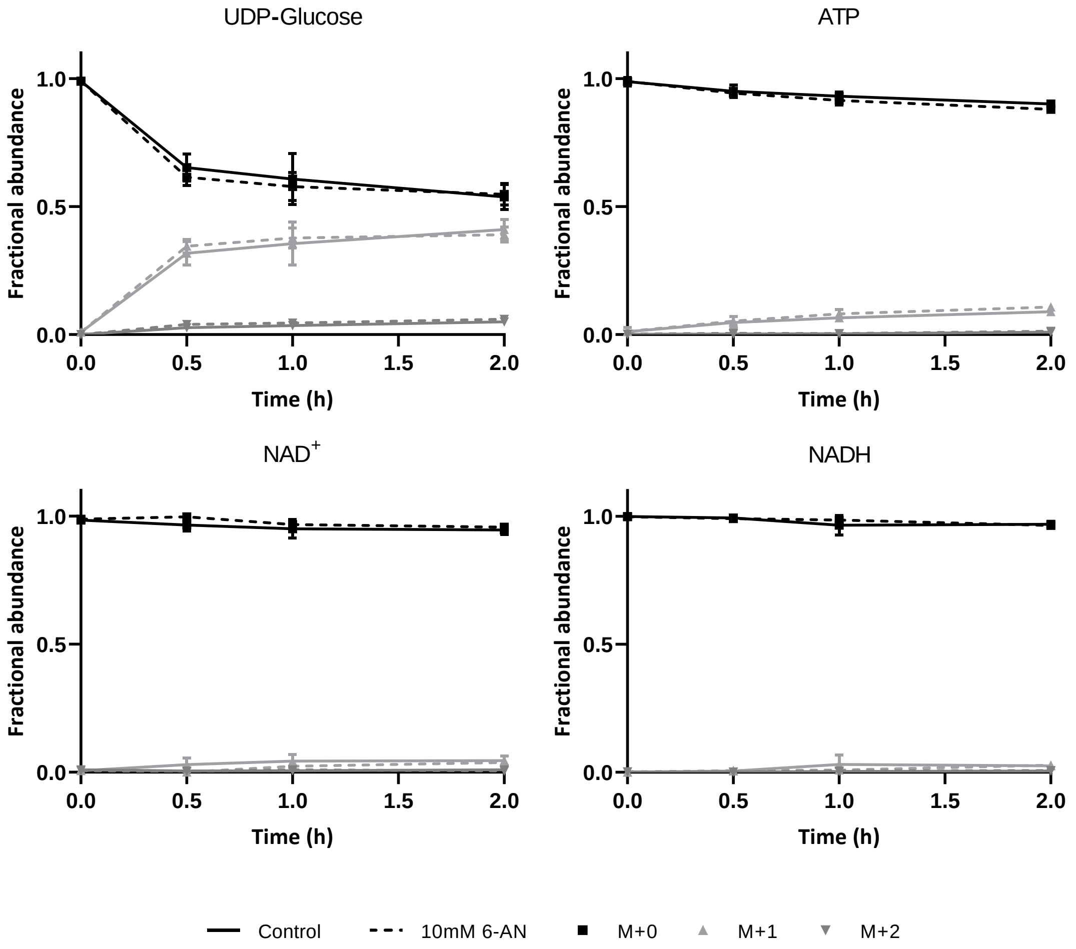

Figure S5. MIDs of metabolites after incubation of *Arabidopsis* cell cultures on 100% [1-^{2}H]glucose media for up to 2 h after incubation with or without 10 mM 6-AN for 20 h. Samples were analysed using Zic-cHILIC chromatography, Xevo G2 XS MS and corrected for natural abundance using IsoCor. Values are the mean ± SD, n = 4.

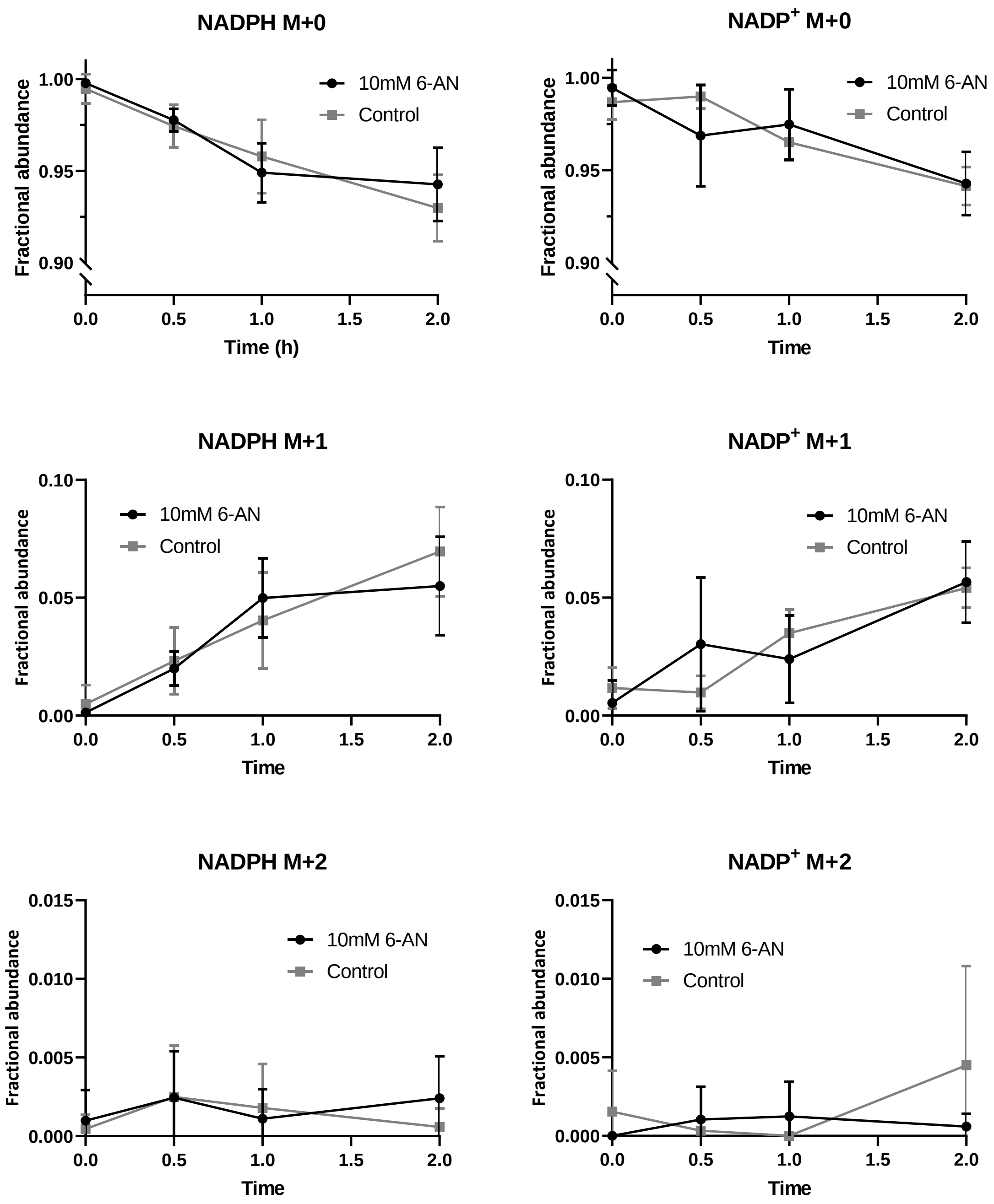

Figure S6. Effect of 10 mM 6-AN on NADP(H) MID following incubation on [1-^{2}H]glucose. Cells were incubated with or without 10mM 6-AN for 20 h prior to transfer to media containing 100% [1-^{2}H]glucose. Values are the mean ± SD of 4 biological replicates corrected for natural abundance. Samples were analysed using Zic-cHILIC chromatography coupled to Xevo G2 XS MS.

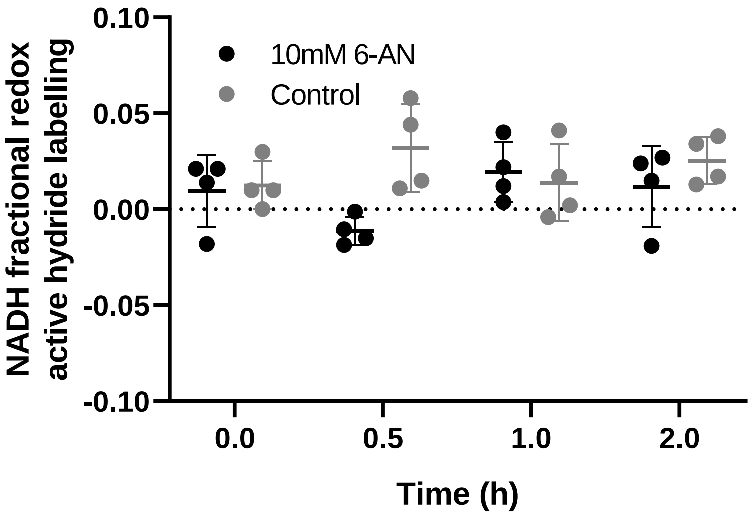

Figure S7. The effect of 6-AN on NADH redox active hydride labelling following transfer to 100% [1-^{2}H]glucose media. Redox active hydride labelling was calculated by deconvolution of NAD^{+} and NADH MID. Cells were incubated with (•) or without(•) 10 mM 6-AN for 20 h before transfer to 100% [1-^{2}H]glucose media and extracted over 2 h. Samples were analysed using Zic-cHILIC chromatography and Xevo G2 XS MS. Each point is a single biological replicate. Error bars are SD.

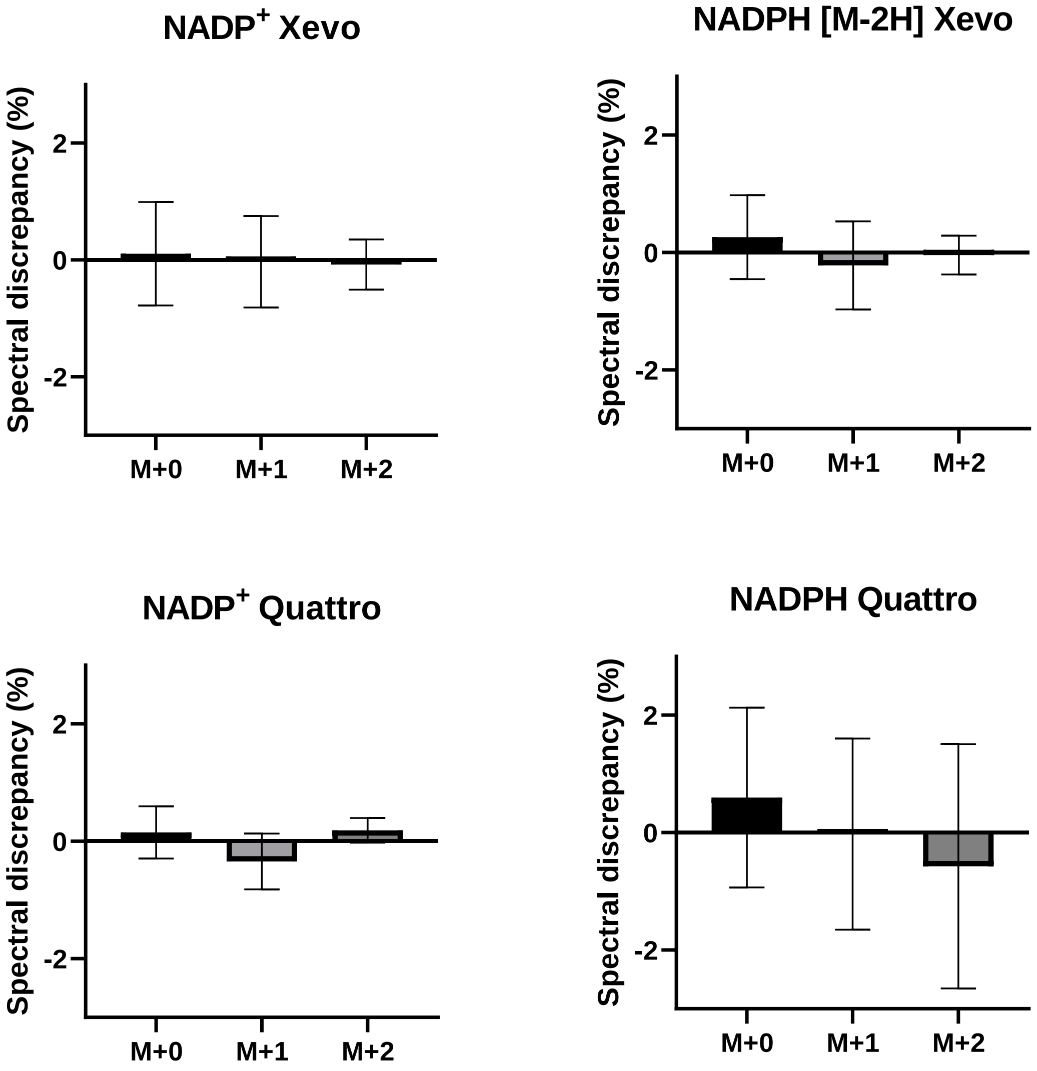

Figure S8. Spectral accuracy of NADP(H) MID measurement from representative biological samples measured using Zic-cHILIC chromatography and Xevo G2 XS or IPRP chromatography and Quattro Micro MS. Spectral discrepancy is the difference between the measured fractional abundance and the predicted fractional abundance from natural abundance or heavy isotopes. Values are the mean ± SD, n ≥ 8.

References

1. Lu, W.; Wang, L.; Chen, L.; Hui, S.; Rabinowitz, J.D. Extraction and quantitation of NAD(P)(H). *Antioxid. Redox Signal.* **2018**, *28*, 167–179.

2. Zhang, Z.; Chen, L.; Liu, L.; Su, X.; Rabinowitz, J.D. Chemical basis for deuterium labeling of fat and NADPH. *J. Am. Chem. Soc.* **2017**, *139*, 14368–14371.

3. Wang, M.; Herrmann, C.J.; Simonovic, M.; Szklarczyk, D.; von Mering, C. Version 4.0 of PaxDb: Protein abundance data, integrated across model organisms, tissues, and cell-lines. *Proteomics* **2015**, *15*, 3163–3168.
